# Prognostic Value of Osteopontin Splice Variant-c Expression in Breast Cancers: A Meta-Analysis

**DOI:** 10.1155/2016/7310694

**Published:** 2016-07-04

**Authors:** Chengcheng Hao, Zhiyan Wang, Yanan Gu, Wen G. Jiang, Shan Cheng

**Affiliations:** ^1^Department of Biochemistry and Molecular Biology, School of Basic Medical Sciences, Capital Medical University, Beijing 100069, China; ^2^Beijing Key Laboratory of Cancer & Metastasis Research, Capital Medical University, Beijing 100069, China; ^3^Cardiff China Medical Research Collaborative, Cardiff University School of Medicine, Heath Park, Cardiff CF14 4XN, UK

## Abstract

*Objectives.* Osteopontin (OPN) is overexpressed in breast cancers, while its clinical and prognostic significance remained unclear. This study aimed to assess the prognostic value of OPN, especially its splice variants, in breast cancers.* Methods.* Data were extracted from eligible studies concerning the OPN and OPN-c expression in breast cancer patients and were used to calculate the association between OPN/OPN-c and survival. Two reviewer teams independently screened the literatures according to the inclusion and exclusion criteria based on quality evaluation. Following the processes of data extraction, assessment, and transformation, meta-analysis was carried out* via* RevMan 5.3 software.* Results*. A total of ten studies involving 1,567 patients were included. The results demonstrated that high level OPN indicated a poor outcome in the OS (HR = 2.22, 95% CI: 1.23–4.00, and *P* = 0.008; random-effects model) with heterogeneity (*I* ^2^ = 62%) of breast cancer patients. High level OPN-c appeared to be more significantly associated with poor survival (HR = 2.14, 95% CI: 1.51–3.04, and *P* < 0.0001; fixed-effects model) with undetected heterogeneity (*I* ^2^ = 0%).* Conclusions.* Our analyses indicated that both OPN and OPN-c could be considered as prognostic markers for breast cancers. The high level of OPN-c was suggested to be more reliably associated with poor survival in breast cancer patients.

## 1. Introduction

Breast cancer is one of the most commonly diagnosed cancers in women worldwide and it is the leading cause of cancer death. According to the statistics provided by the American Cancer Society, approximately 231,840 new cases of invasive breast cancer and 40,290 breast cancer deaths occurred among US women in 2015 [1]. It was estimated by GLOBOCAN 2008 that breast cancer was the most frequent cancer in Chinese women, with an age standardized rate (ASR) of 21.6 cases per 100,000 individuals [2]. Although multiple treatment methods including surgery, chemotherapy, radiotherapy, and targeted therapy have been applied over the past few decades, the prognosis of breast cancer remains unsatisfactory. Due to the limitations in prognostic values of the conventional predicting factors, such as TNM stage, age, sex, and histological type, it is necessary to explore novel biomarkers to better predict the outcome and assist in the clinical management of breast cancer patients.

Osteopontin (OPN), a phosphorylated glycoprotein secreted by various tissues and cells, has been implicated with important roles in physiological and pathological processes [13–15]. In recent years, accumulating evidences have shown that aberrant OPN expression was closely associated with tumorigenesis and metastasis in several types of tumors, including breast cancer [16, 17]. Overexpression of OPN was found in multiple malignant breast cancer cell lines, and the transfection of OPN into benign breast epithelial cells induced invasive behavior [18]. In addition, several studies investigated the association between OPN expression and the clinical outcome and prognosis of breast cancer patients, but the results failed to demonstrate a consented conclusion regarding the ability of OPN to predict cancer progression [7, 19–21].

The biological functions of metastasis-associated gene products are often mediated by different splicing isoforms. Alternative splicing OPN secreted by various cells has diverse structural characteristics. Tumor-derived OPN forms are smaller than OPN secreted by nontransformed cells. Full length OPN (OPN-a) consists of 7 exons while OPN-b and OPN-c lack exon 4 and exon 5, respectively [22, 23]. Alternative splicing occurred at the upstream of the central integrin binding domain and the C-terminal CD44 binding domain [24–26]. Clinical data suggested that the shortest splice variant OPN-c could be a selective diagnostic and prognostic candidate for human breast cancer [27–29]. However, the clinical function of OPN-c in breast cancer remains poorly defined.

Therefore, to clarify the prognostic significance of OPN, as well as its splicing variants, in breast cancer, it is necessary to investigate the association of OPNs expression with patient survivals. In the present study, we pooled data from the available reports and analyzed the association between the expression of OPN and the prognostic measures of breast cancer patients.

## 2. Materials and Methods

### 2.1. Study Selection

A comprehensive literature search (dated to December 2015) was conducted through the PubMed database. The query strategy was the combinations of the following terms: “Osteopontin”, “OPN”, “OPN-c”, “Osteopontin-c”, “breast cancer”, “prognosis”, “prognostic” and “survival” without restrictions on regions, languages, and publication types. All eligible studies were retrieved.

### 2.2. Inclusion and Exclusion Criteria

The criteria for reference inclusion were (1) data associated with OPN levels (negative/positive or low/high expression), as measured by reverse transcription-polymerase chain reaction (RT-PCR), immunohistochemistry (IHC), or enzyme linked immunosorbent assay (ELISA); (2) confirmed diagnosis of breast cancer with pathological or histological evidence; (3) hazard ratio (HR) and their 95% confidence intervals (CIs) that could be directly extracted or disease-free survival or overall survival with sufficient data that was provided; (4) some data supplied by authors upon our requests for the calculation of the HR or 95% CIs values.

The studies were excluded for (1) meeting abstracts, letters, case reports, and reviews; (2) being animal studies; (3) missing necessary data; and (4) using redundant or overlapped database records.

### 2.3. Data Extraction and Quality Assessment

To reduce bias and improve reliability, two investigator groups independently reviewed potentially relevant studies. The data of the number of patients, follow-up, detection method, and cut-off value, together with name of the first author, name of the journal, year of publication, and ethnicity, were extracted and then analyzed against OPN/OPN-c expression-related survival. Conflicting interpretation problems were resolved through consensus with the third investigator. The outcome assessment focused on survival curves in patients with different OPN/OPN-c expression. The GetData Graph Digitizer 2.24 software (http://getdata-graph-digitizer.com/) and HR digitizer Engauge 4.1 software (http://engauge-digitizer.software.informer.com/) were used to digitize and extract the data from the Kaplan-Meier curves, in cases when only survival curves were provided. The quality of each included study was based on two independent assessments by Newcastle-Ottawa Scale (NOS) scoring. We allocated a score of 0–9 to each included study, and those with a NOS score ≥ 6 were assigned as high-quality studies.

### 2.4. Statistical Analysis

The association between OPN/OPN-c expression and the survival outcome of breast cancer was estimated according to the HR and 95% CI directly or indirectly extracted from each eligible study. The heterogeneity among studies was detected using Cochran's *Q* test and Higgins *I*-squared statistic. Severe heterogeneity was taken into account based on measures of *I* ^2^ > 50%. The fixed-effects model was used when *I* ^2^ < 50% or *P* ≥ 0.10 in the *Q* test, while random-effects model was conducted otherwise. The potential publication bias was estimated by Egger's and Begg's tests with significance of *P* < 0.05. All the statistical analyses were performed using Review Manager version 5.3.

## 3. Results

### 3.1. Characteristics of Included Studies

**Figure 1:**
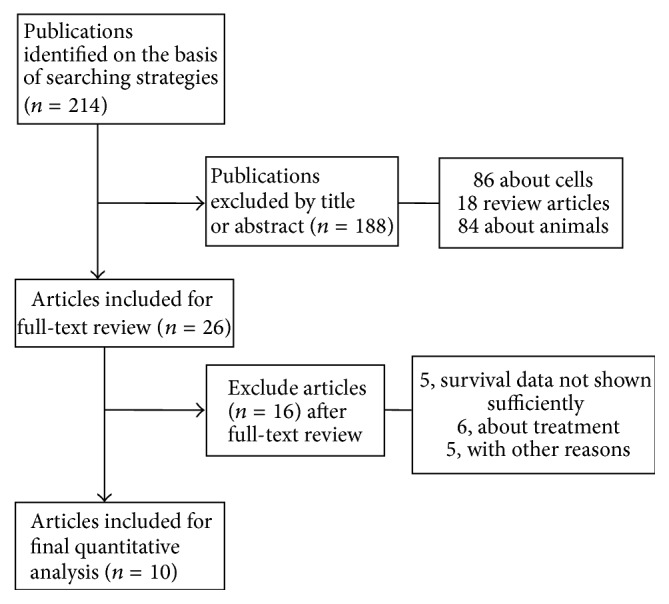
Flowchart of the selection process of studies for inclusion in this meta-analysis.

Following the search strategy given in Section 2, a total of 214 potentially relevant citations were retrieved (Figure 1). After reviewing the titles and abstracts, 188 studies failed to meet our selection criteria and were excluded. The remaining 26 were subjected to full-text screening, of which 16 publications were excluded because of the lack in survival or follow-up data associated with OPN/OPN-c. Eventually, a total of 10 studies including 1,567 patients were qualified for further analysis, 7 of which investigated the impact of OPN expression on survival and 4 were about OPN-c. Three of the 10 studies used ELISA, 5 used IHC, and 2 used qRT-PCR to detect serum- or tissue-derived OPN/OPN-c expression. Detailed values from the included publications were summarized in Table 1.

### 3.2. Association of OPN on OS of Breast Cancer Patients

**Figure 2:**
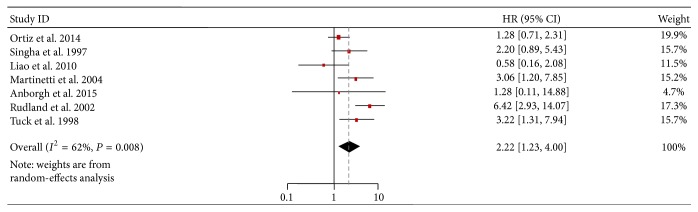
Forest plot demonstrating the association of OPN expression with overall survival in breast cancer. HR, hazard ratio; CI, confidence interval.

Seven included studies provided data for calculating the association between OPN expression and survival. We pooled all the available data into our meta-analysis and found that poor OS was significantly associated with a positive status for OPN (HR = 2.22, 95% CI: 1.23–4.00, and *P* = 0.008) (Figure 2), with heterogeneity in the data (*I* ^2^ = 62%, *P* = 0.01).

**Figure 3:**
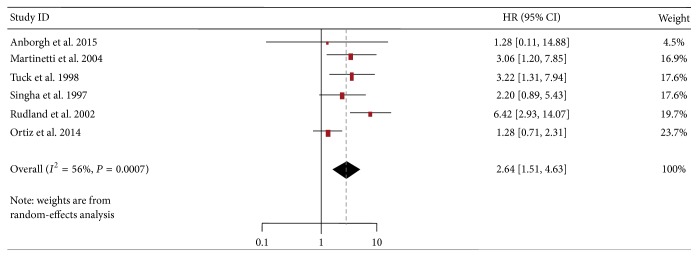
Forest plot on the association between OPN expression and overall survival in breast cancer by sensitivity analysis. HR, hazard ratio; CI, confidence interval.

To determine whether the heterogeneity in OS was caused by data bias, we performed sensitivity analysis to assess the stability of the results. The results showed that, after exclusion of the study by Liao et al., the heterogeneity for OS did not significantly decrease (*I* ^2^ = 56%, *P* = 0.0007) with a combined HR of 2.64 (95% CI: 1.51–4.63) (Figure 3). The fact that the HR and *I* ^2^ values were not significantly altered with the exclusion of distracted dataset indicated that the method was appropriate and the results were credible.

**Figure 4:**
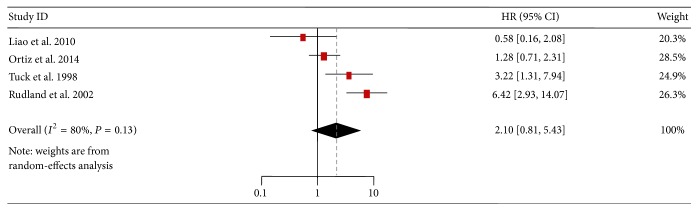
Forest plot demonstrating the association of OPN expression with overall survival in tissues of breast cancer patients. HR, hazard ratio; CI, confidence interval.

**Figure 5:**
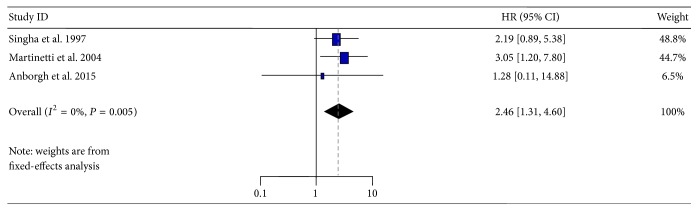
Forest plot demonstrating the association of OPN expression with overall survival in blood of breast cancer patients. HR, hazard ratio; CI, confidence interval.

The subgroup meta-analyses were performed to exclude the potential influence from sample heterogeneity. Four studies (three using IHC, one by qPCR) were included to evaluate the connection between OPN expression in breast tumor tissues and survival. Positive OPN expression was associated with poor OS (HR = 2.10, 95% CI: 0.81–5.43) with heterogeneity (*I* ^2^ = 80%, *P* = 0.002), but no significant difference (*P* = 0.13) was found (Figure 4). Three studies about the association between plasma OPN level and survival were also investigated. Results showed that reduced survival was significantly associated with a positive status for plasma OPN (HR = 2.46, 95% CI: 1.31–4.60, and *P* = 0.005) (Figure 5), with diminished heterogeneity in the data (*I* ^2^ = 0%, *P* = 0.76).

### 3.3. Association of OPN-c on Survival of Breast Cancer Patients

**Figure 6:**
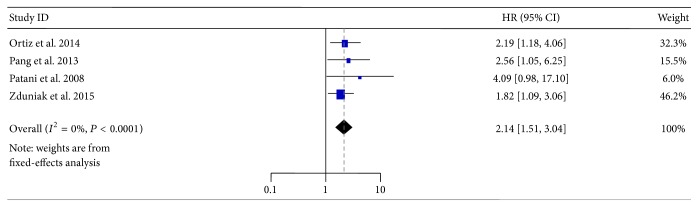
Forest plot demonstrating the association of OPN-c expression with overall survival in breast cancer. HR, hazard ratio; CI, confidence interval.

Four studies (two OS, one on DFS, and one with five-year survival) including 853 patients were investigated. The statistical results once again indicated that high OPN-c expression in tissues was significantly associated with poor survival (HR = 2.14, 95% CI: 1.51–3.04, and *P* < 0.0001) with diminished heterogeneity (*I* ^2^ = 0%, *P* = 0.72) (Figure 6).

### 3.4. Publication Bias

**Figure 7:**
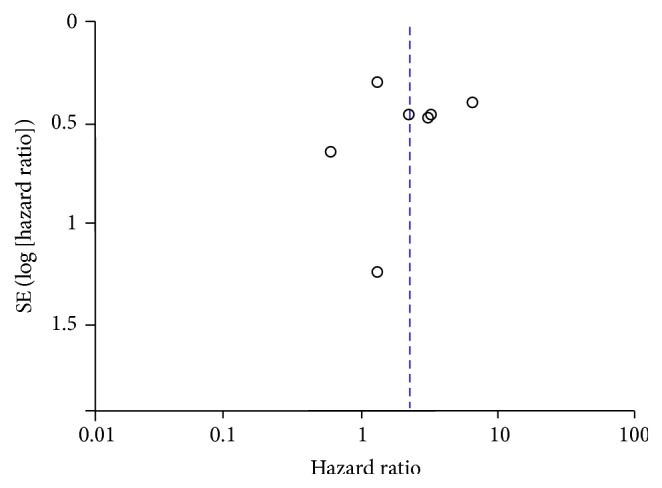
Begg's funnel plot estimating the publication bias of the included studies about OPN. HR, hazard ratio.

**Figure 8:**
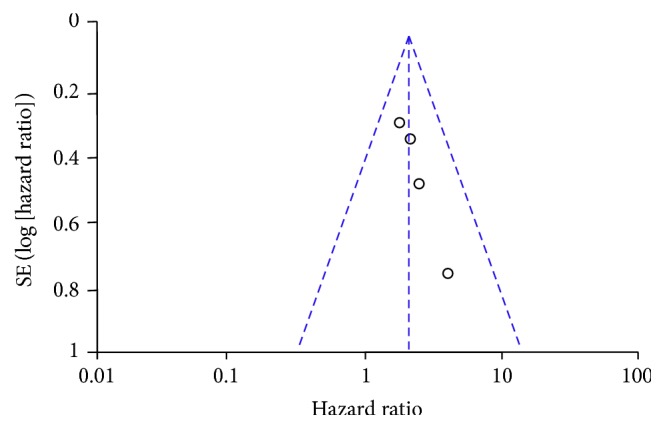
Begg's funnel plot estimating the publication bias of the included studies about OPN-c. HR, hazard ratio.

In order to assess the publication bias of the included studies, Begg's funnel plot was deployed. As shown in Figures 7 and 8, nonasymmetric funnel plots for the synthesis of the HRs for survival were obtained. Hence, no publication bias was detected among all the comparisons.

## 4. Discussion

As a popular and effective approach for systematic reviews, meta-analysis has been successfully applied to evaluate prognostic indicators in patients with varied diseases. It has been indicated in recent research that the expression of biomarkers was promisingly associated with tumor progression, including breast cancers. Besides, its elevated levels often suggested high tumor grade, metastasis, and poor prognosis. Several markers have been used in breast cancer diagnosis, including estrogen receptor (ER) and progesterone receptor (PR). However, these markers still lack the adequate sensitivity and specificity for detecting prognosis of breast cancer patients. It was reported that OPN might be a more potential clinical marker candidate for predicting the survival of breast cancer patients than ER and PR [6, 7, 9]. A meta-analysis was further performed in our group to clarify the above ambiguous conclusion and to investigate the association of OPN with prognostic factors in breast cancer.

From a number of reports, OPN was overexpressed in primary tumors and maintained a high secreted level in the blood of breast cancer patients, which correlated with a poor prognosis [3–6, 8, 9, 30]. The results of our present meta-analysis study basically supported the association between increased OPNs with poor OS in patients with breast cancer (*P* = 0.008). However, there could be a concern with the heterogeneity (*I* ^2^ = 62%). Through the sensitivity analysis, the resulting heterogeneity value (*I* ^2^ = 56%) without substantial changes indicated an intrinsic property and would not compromise the robustness and prognostic values of OPN in breast cancer. The heterogeneity problem was also reported in a recent similar meta-analysis conducted by Xu et al., which explored the prognostic value of OPN by OS and DFS in breast cancer patients. Notably, the further subgroup analysis showed that the increased OPN was not correlated with clinicopathological parameters such as tumor grade, tumor stage, PR status, ER status, and p53 status, suggesting that the source of heterogeneity could be rather complicated [31]. The prognostic value of OPN has been reported in systematic reviews and meta-analyses on lung [32], colorectal [33], and pancreatic [34] cancers with a vast range in heterogeneities and the positive association of increased OPN with the survival of cancer patients appeared to be consistent, despite the fact that certain additional factors were indeed to be considered for the statistical analysis of heterogeneity and its origins.

Alternative RNA splicing of human OPN results in three transcriptive variants: OPN-a (full length), OPN-b (lacking exon 5), and OPN-c (lacking exon 4). Recent studies have shown that cell-type specific expression of OPN splice variants exerted different functions in malignant tumors. Compared to full length OPN, OPN-c was specifically expressed in breast cancer cells. Hence, it was likely to be a better prognostic marker for human breast cancer [9–12]. Unfortunately, studies have not conducted separate measures on OPN splice variants. The results were actually the total OPN (including OPN-a, OPN-b, and OPN-c) expression in plasma or tumor of breast cancer patients. The only study that can be found reported that the survival for breast cancer patients with high levels of OPN-b mRNA expression was found to differ significantly from that of their low level counterparts [12]. The available data of OPN-c for cancer survival only allowed primitive comprehensive analysis. In the present study, we carried out a meta-analysis and noted that the high level of OPN-c was associated with poor survival with better statistical significance (*P* < 0.0001) and, most of all, with a drastically reduced heterogeneity (*I* ^2^ = 0%). Thus, OPN-c could be by far a most significant predictor of the poor prognosis of breast cancers, which could be further investigated in studies involving more breast cancer patients or perhaps in other cancer types as well.

Interestingly, in the subgroup meta-analysis, positive OPN expression in breast tumor tissues was associated with poor OS, but no significant difference (*P* = 0.13) was found (Figure 4), while reduced survival was significantly associated with a positive status for OPN in plasma (*P* = 0.005) (Figure 5). These results seemed to be contrary; however, they were still considered to confirm the results that the total tumor OPN expression was very heterogeneous. As a more significant marker candidate, OPN-c is likely to be of particular utility as a prognostic marker and should be included in further validation studies. The significant association between plasma OPN and survival suggested that the plasma detection could be a powerful supplement to the analysis of tumor tissues. However, there were no reports about the association between plasma OPN-c and the prognosis of breast cancers till now. Therefore, it is very important and necessary to perform the corresponding studies.

We have recognized that there are several limitations existing in this meta-analysis. First, the literatures covering this topic are limited in numbers. The database documenting their related data is small in scale, especially those about OPN-c. Second, the investigation regarding the correlations between OPN/OPN-c expression and clinical features, such as tumor stage, lymph node metastasis, and tumor size, is not performed, because most of the primary studies have not provided sufficient information for this analysis. Third, different methods are adopted and inconsistent cut-off values are used for determining the expression of OPNs in different reports, and therefore it is a challenge for the normalization of the results. Fourth, in dealing with the reports which do not contain the HRs in the full text, we extrapolated the values from the survival curves. Although this approach is a common practice, we cannot exclude the possible introduced errors. Finally, to avoid the potential publication bias, factors, such as sex, age, and geographical origins, are not considered in this analysis.

In summary, both OPN and OPN-c could be considered as markers for breast cancer prognosis. The finding of better performance of OPN-c in statistics suggested that the stratification of different isoforms or variants of gene expression could be a valid approach for searching better biomarkers in cancer patients. Based on the findings from this study, we recommend a prospective study to confirm the prognostic value of OPN-c in breast cancer patients.

## Figures and Tables

**Table 1:** General characteristics of included studies.

	First authors	Journal	Year	Country	Number of patients	Follow-up (months)	Index	Methods	Cut-off point (high/low)	OPN+/OPN−	HR estimation	Quality assessment (0–9)
1	Anborgh [3]	Am J Transl Res	2015	UK	53	76	OPN	ELISA	Median	31/22	HR + 95% CI	7
2	Martinetti [4]	Endocrine-Related Cancer	2004	Italy	34	60	OPN	ELISA	Median	12/22	Survival curves	8
3	Singhal [5]	Clin Cancer Res	1997	Canada	70	19	OPN	ELISA	>203	24/46	Survival curves	7
4	Tuck [6]	Int. J. Cancer	1998	Canada	154	195.6	OPN	IHC	Score > 4	11/143	Survival curves	5
5	Rudland [7]	Cancer Res	2002	UK	333	228	OPN	IHC	>5%	221/112	RR + 95% CI	8
6	Liao [8]	Modern Oncology	2010	China	70	110	OPN	IHC	Score ≥ 2	51/19	Survival curves	5
7	Ortiz-Martínez [9]	Human Pathology	2014	Spain	309	302	OPN/OPN-c	qRT-PCR	FC > 32	76/231 70/204	DFS + OS	7
8	Pang [10]	Cancer Epidemiology	2013	China	170	92	OPN-c	IHC	Score ≥ 2	119/51	HR + 95% CI	6
9	Zduniak [11]	British Journal of Cancer	2015	USA	291	60	OPN-c	IHC	Score ≥ 2	181/110	Survival curves	7
10	Patani [12]	Anticancer Research	2008	UK	83	150	OPN-c	qRT-PCR	NPI	21/62	OS	6
